# Personalized monotherapy vs. protocol therapy complex for frozen shoulder. Comparative study

**DOI:** 10.1186/1878-5085-5-S1-A163

**Published:** 2014-02-11

**Authors:** Rostyslav V Bubnov, Andrii M Strokan, Petro V Logvinchuk, Tetiana S Aleksieieva

**Affiliations:** 1 Ultrasound, Clinical Hospital ”Pheophania“ of State Affairs Department, Kyiv, Ukraine; 2 Anesthesiology, Clinical Hospital ”Pheophania“ of State Affairs Department, Kyiv, Ukraine; 3 Neurology, Clinical Hospital ”Pheophania“ of State Affairs Department, Kyiv, Ukraine

## Background

Many approaches exist for frozen shoulder, rotators myofascial trigger points (MTrP) inactivation is crucial therapeutic point [1,2].

## The aim

was to determine efficacy of additional treatment after MTrP inactivation.

## Materials and methods

We included 30 patients, 13 males 17 females, aged 42-63 years (the average was 54 years) with diagnosed frozen shoulder, MTrP were identificated in rotator muscles (infraspinatus, supraspinatus, subscapular and teres minor muscles). Patients were randomly assigned to: group A - MTrP dry needling (DN) under ultrasound guidance; patients of group B additionally received conservative treatment (physiotherapy, massage, gymnastics) according to accepted treatment protocol. All patients had symptoms over 1 month, underwent general diagnostic examination including MRI, laboratory, neurologic, orthopedic tests, neuropathy, spine diseases were excluded. Visual analogue scale data (VAS, 0-10); Disabilities of the Arm, Shoulder and Hand (DASH) disability, Subjective global function (0-100) scores were measured before, immediately after, 24 hours, 14 days after intervention. We evaluated pain and trigger point (spasticity) recurrence 24 hours and 14 days after manipulation in both groups.

## Results

After 14 days, VAS shown pain improvement from 7.3 to 1.1 in group A compared to 7.4 to 4.5 in group B (P < 0.01); DASH scores improved by 40 % (161.3 to 113.4) in A vs. 25% (131.26 to 84.7) in group B (P < 0.05); Subjective global function scores, improved from 56 and 58 at baseline to 73, 91 respectively (P < 0.05). MTrP recurrence was lower in group A: 30% vs. 53% in group B (P < 0.01) at 24 hours after manipulation; outcome at 14 day was 7% vs. 27% respectively (P < 0.05).

## Conclusions

DN under Ultrasound guidance of MTrP in rotators is effective method for frozen shoulder, is preferred as personalized monotherapy for pain relief and prevent trigger point (spasticity) relapse.

## Outlook and expert recommendations

The larger cohort studies (preferable multicenter RCT) are recommended to start to establish science based treatment algorithm.
